# Landscape Mapping of Functional Proteins in Insulin Signal Transduction and Insulin Resistance: A Network-Based Protein-Protein Interaction Analysis

**DOI:** 10.1371/journal.pone.0016388

**Published:** 2011-01-31

**Authors:** Chiranjib Chakraborty, Sanjiban S. Roy, Minna J. Hsu, Govindasamy Agoramoorthy

**Affiliations:** 1 School of Bioscience and Technology, VIT University, Vellore, India; 2 School of Computing Science and Engineering, VIT University, Vellore, India; 3 Department of Biological Sciences, National Sun Yat-Sen University, Kaohsiung, Taiwan Authority; 4 College of Environmental and Health Sciences, Tajen University, Yanpu, Taiwan Authority; King Abdullah University of Science and Technology, Saudi Arabia

## Abstract

The type 2 diabetes has increased rapidly in recent years throughout the world. The insulin signal transduction mechanism gets disrupted sometimes and it's known as insulin-resistance. It is one of the primary causes associated with type-2 diabetes. The signaling mechanisms involved several proteins that include 7 major functional proteins such as INS, INSR, IRS1, IRS2, PIK3CA, Akt2, and GLUT4. Using these 7 principal proteins, multiple sequences alignment has been created. The scores between sequences also have been developed. We have constructed a phylogenetic tree and modified it with node and distance. Besides, we have generated sequence logos and ultimately developed the protein-protein interaction network. The small insulin signal transduction protein arrangement shows complex network between the functional proteins.

## Introduction

The occurrence of type 2 diabetes, especially in the developing countries has increased rapidly during the last decade. The International Diabetes Federation has estimated that the number of diabetic patients in India alone more than doubled from 19 million in 1995 to 40.9 million in 2007. It is projected to increase to 69.9 million by 2025. Presently, 285 million people throughout the world are living with diabetes and a vast majority (90%) is caused by the type 2 diabetes [1]. The damage of insulin signal transduction causes insulin resistance, which is the main causation for the type 2 diabetes. It has been predicted that the type 2 diabetes will increase 2.5 times in the Middle East, Sub-Saharan Africa, Latin America, India, and rest of Asia during 21st century therefore this diseases has the potential to become a new epidemic globally [2]–[3].

Various proteins are associated with the signal transduction of insulin of which the major ones are insulin receptor, insulin receptor substrate 1, insulin receptor substrate 2, type 1A phosphatidylinositol 3–kinase, Akt2 protein, and glucose transporter type [4]. Insulin receptor is one of the main proteins for signaling mechanism and it consists of two extracellular α subunits and two transmembrane β subunits. Alterations in insulin receptor expression, binding, phosphorylation state, and/or kinase activity could account for many insulin resistances [5]–[6]. Insulin receptor substrate (IRS) proteins are the key moderators of insulin action since they regulate downstream signaling and protein-protein interactions in the biochemical pathway [6]. In addition, several studies have proposed insulin-stimulated phosphatidylinositol 3–kinase (PI 3-kinase) in view of the fact that it plays an important role in the regulation of insulin-mediated glucose uptake in human. Furthermore, Akt-kinase, also known as PKB (protein kinase B), demonstrated as a key protein in the insulin signaling pathway linking the activation of PI 3-kinase to glucose uptake [7]–[8]. Understanding the protein-protein interaction in this particular signaling pathway could lead to comprehend the significance of insulin resistance better.

Mapping the protein-protein interaction is an essential step towards the framework of biological signaling pathway [9]. The protein-protein interaction represents mutual relationship between the proteins and the mapping of such interactions can be predicted by high-throughput proteomic analysis or links based on protein components between pathways and complexes [10]. With the advancement of computer science technology, mapping proteins onto signaling pathways could be easily and rapidly developed. Using such computational methods, large-scale proteomics information can be analyzed thoroughly. In this way, we can immunize experiment numbers required to detect the main components of a pathway [11]. Several protein networks database for biochemical pathway have already been developed to expand the knowledge of protein-protein interactions and some notable examples include the reference databases involving human protein interactions and signaling pathways [12]–[15].

In this paper, we have applied computational biology to map out the protein-protein interaction. We have highlighted 7 principal proteins such as INS, INSR, IRS1, IRS2, PIK3CA, Akt2, and GLUT4 in insulin signaling pathway and we have collected data on their genes from the global databases. Using the 7 primary proteins, we have carried out multiple sequences alignment to develop scores between sequences. We have successfully constructed phylogenetic tree and also modified phylogenetic tree with node and distance. Besides, we have generated sequence logos and ultimately introduced the protein-protein interaction network. The small insulin signal transduction protein network presented in this paper shows a complex network pattern among the functional proteins.

## Materials and Methods

### Collection of data

The first step toward a protein interaction network-based modeling of insulin signaling pathway involves listing of human proteins and related genes. We have identified 7 principal proteins (INS, INSR, IRS1, IRS2, PIK3CA, Akt2, GLUT4) in insulin signaling pathway and collected information about their genes from the database. The functional protein sequences in FASTA format for these genes were collected from the National Center for Biotechnology information (www.ncbi.nih.nlm.gov). The functional protein sequences in FASTA for these genes were used for further analysis.

### Multiple sequences alignment and phylogenetic tree construction

We have used ClustalW (www.ebi.ac.uk/clustalw), a general purpose multiple sequence alignment computer software for proteins, which produces biologically meaningful multiple sequence alignments of divergent sequences. It calculates the best match for sequences, and lines them up so that the identities, similarities and differences can be observed and analyzed. Evolutionary relationships can also be observed by viewing Cladogram or Phylogram. Using the multiple sequence alignment technique, we have observed the similarity in the sequences and their respective alignment scores were elucidated. In this analysis, seven sequences were used (INS, INSR, IRS1, IRS2, PIK3CA, Akt2, GLUT4 sequences represent as Seq1, Seq2, Seq3, Seq4, Seq5, Seq6, Seq7, respectively). We have used notation Seq (x:y) meaning alignment score between sequence x, and sequence y. The scores were further used for analysis. Based on these results of the sequence alignments, phylogenetic tree was constructed [16]. We have also developed a phylogenetic tree (phylogram) to show the distances between protein sequences involving insulin resistance pathway.

### Modified phylogenetic tree, 3D structure of proteins and sequence logos

We have used MATLAB (7.3 version) programming to depict the modified phylogenetic tree with distance. The tree was constructed using the distance between the nodes of the phylogenetic tree. Furthermore, we have developed an algorithm for the generation of this tree. The ribbon structure regarding the proteins used in this study was collected from the Protein Data Bank [17]. We have pooled data for 6 proteins to generate a sequence logo by using WebLogo software for graphical representation of amino acid or nucleic acid for displaying the patterns in a set of aligned sequences [18]–[19]. We have also used the method to visualize patterns of aligned sequences as well as the bias amino acid sequences in functional protein.

### Protein-protein network design

Using STRING (http://string-db.org), a database of known and predicted protein interactions, we have developed the protein-protein interaction in insulin resistance in signaling pathway. This web-based database dedicated to protein-protein interactions that include direct (physical) and indirect (functional) associations [20].

## Results and Discussion

**Figure 1:**
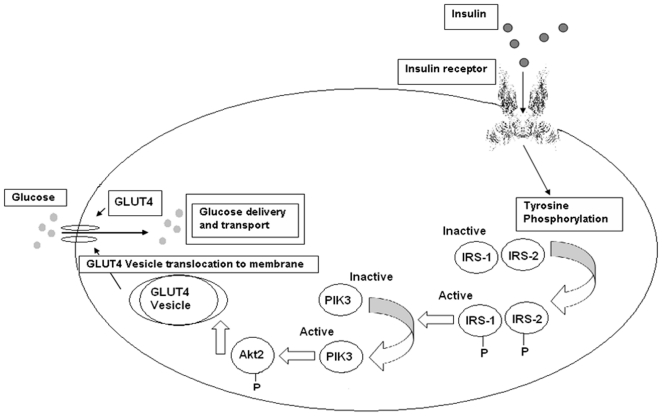
Insulin signal transduction pathway. The damage of insulin signal transduction causes insulin resistance, which is one of the main causes for type 2 diabetes.

**Figure 2:**
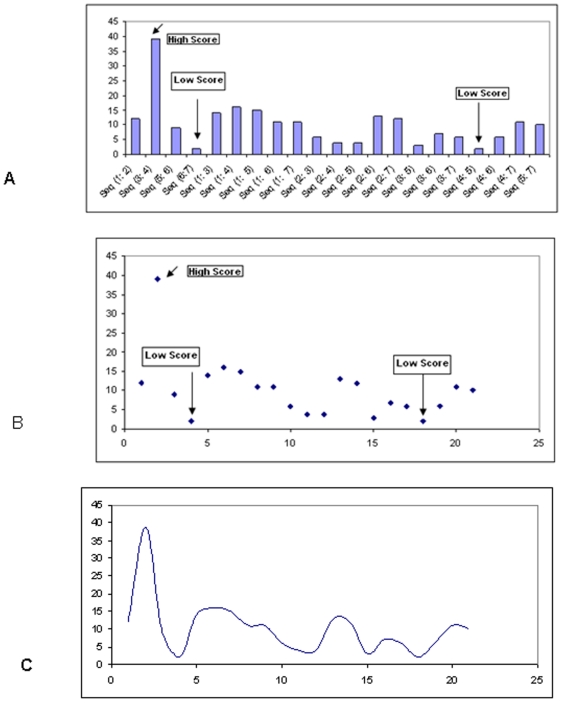
Alignment scores of protein sequences related to insulin resistance pathways. (A) Alignment score between sequences (notation Seq (x:y) meaning alignment score between sequence x, and sequence y), (B) scatter distribution of scores, and (C) scores connected by smoothed line without marker.

**Figure 3:**
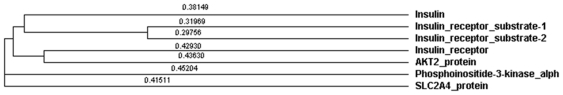
Phylogenetic tree construction using ClustalW.

**Figure 4:**
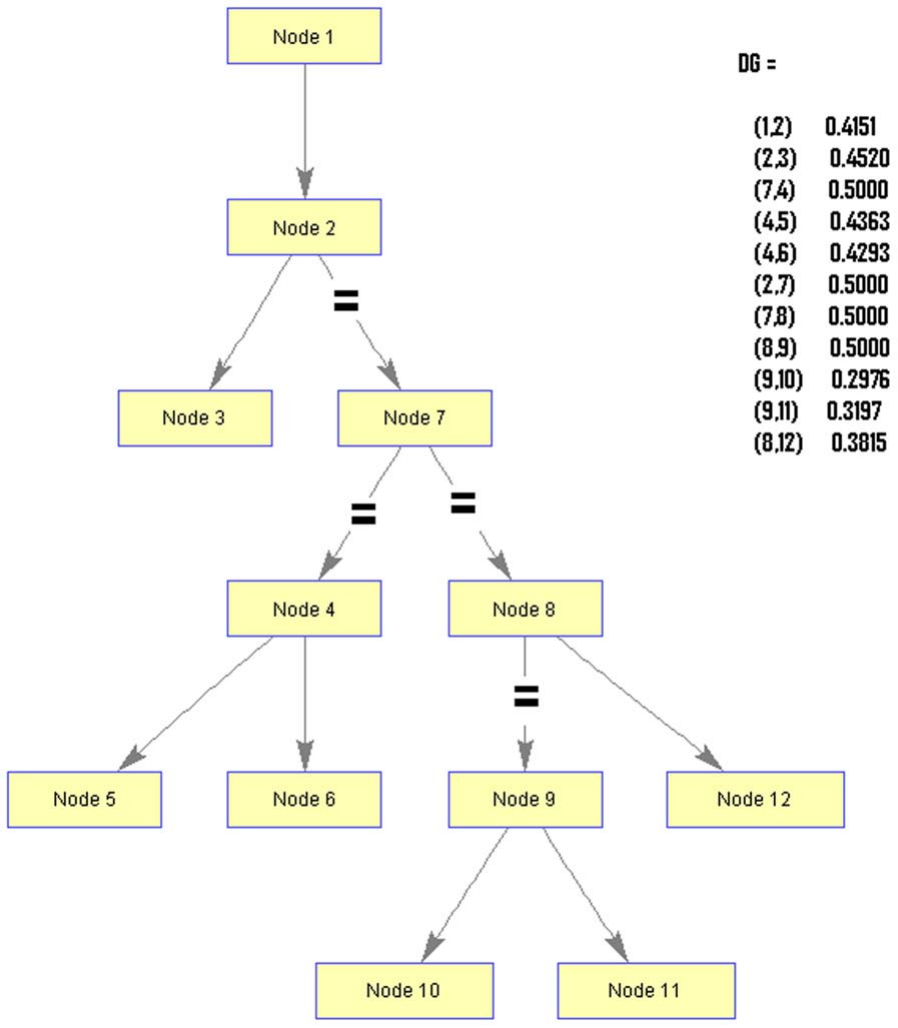
Distance of the phylogenetic tree of 12 nodes (developed by MATLAB 7.3).

Insulin signaling pathways related to functional proteins and their genes were recorded after pooling data from the archives of the NCBI databank (Figure 1). Human functional proteins related to insulin signaling and resistance pathway and their protein ID, accession number, GI and length of the protein were documented. The sequence alignment scores between the sequences were illustrated in Figure 2. The sequence alignment shows highest scores (39) between the sequences 3 and 4. The result not only signifies the sequence matching between IRS1 and IRS2 but also shows the best sequence matching. Lowest scores (02) were observed between the sequences 4 and 5 as well as sequences 6 and 7, respectively. Hence we have observed a vast dissimilarity between the IRS2 and PIK3CA sequence as well as Akt2 and GLUT4 sequence. The constructed phylogenetic tree shown in Figure 3 indicated that IRS1 and IRS2 have similar origins. Similarly, INSR and Akt2 have also analogous origins. Furthermore, we have depicted another phylogenetic tree entitled “modified phylogenetic tree with node and distance” shown in Figure 4 by using a program as follows:

W =  [.41511 .45204 .50 .50 .4363 .4293 .50 .50 .29756 .31969 .38149];

DG  =  sparse ([1 2 2 7 4 4 7 8 9 9 8],[2 3 7 4 5 6 8 9 10 11 12],W)

We have used this program for MATLAB (7.3 version) and also used sparse matrix of our phylogram. The MAT LAB program is as follows:

UG  =  sparse ([1 2 2 7 4 4 7 8 9 9 8],

[2 3 7 4 5 6 8 9 10 11 12], true, 12, 12)

View (biograph(UG,[]))

**Figure 5:**
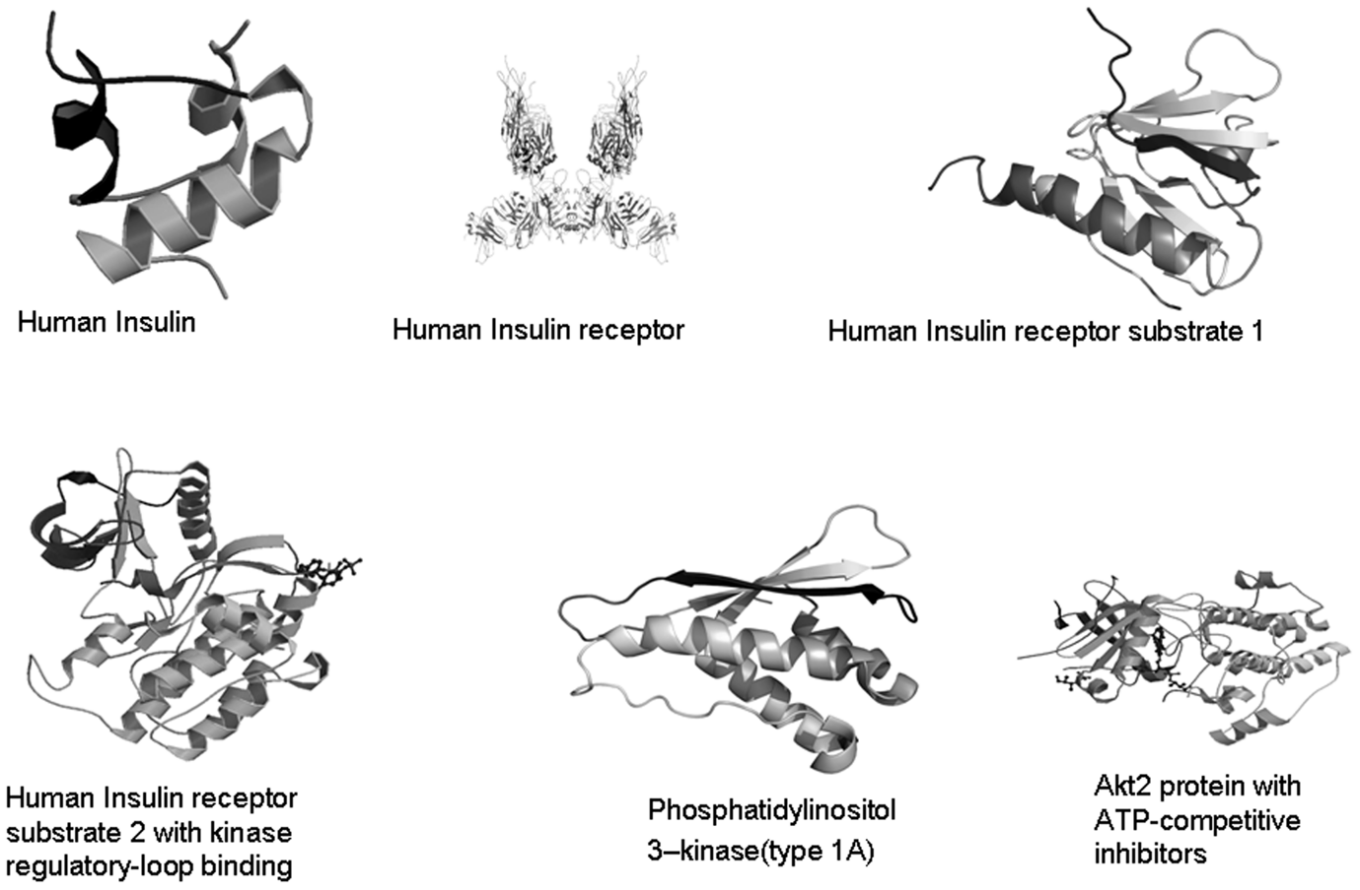
Ribbon like structure of the main functional proteins associated with pathway. Collected from Protein Data Bank.

**Figure 6:**
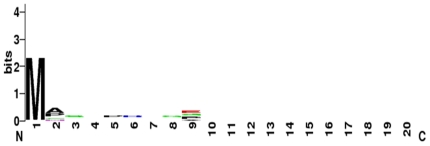
Main proteins associated with insulin signaling pathway. Using WebLogo.

**Figure 7:**
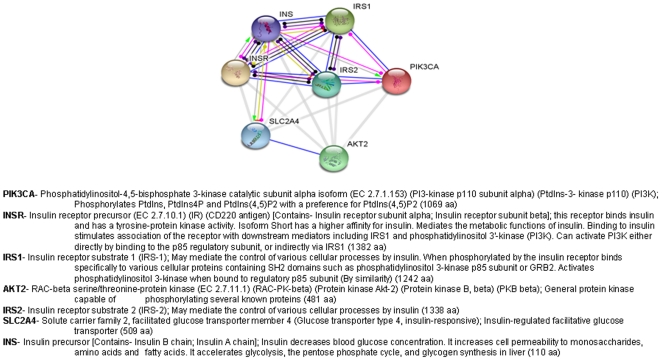
Protein-protein network in insulin signal transduction pathway.

There were 12 nodes in the tree and unknown distance was mapped as .50. We have collected 6 major protein ribbon structures form the protein data bank. Ribbon diagram of the structures have shown alpha helices and beta sheets of six functional proteins (Figure 5). The graphical representation of amino acid for functional proteins was generated through WebLogo shown in Figure 6. The protein-protein network in insulin signal transduction pathway has been shown in Figure **7**.

The insulin signaling mechanism is complex and highly integrated network that relates to several proteins and downstream regulation of proteins. Taniguchi et al. [21] proposed ‘critical nodes’ to define the important junctions for insulin signal path way. However, it appears that several nodes are involved in this signaling process [21]. In this study, we were able to map out a simple protein-protein interaction network between the major seven functional proteins using computational biology. In fact, the protein interaction networks represent mutual relationships between the proteins. For example, protein-A binds to protein-B, then protein-B binds to protein-C or similar way, and this type of representation often applies to predictions made by high-throughput proteomic analysis, or protein components between pathways and complexes [10]. The insulin signals transduction protein network that we have presented in this paper shows a complex system between functional proteins. It also shows a strong protein network relationship between proteins like INS, INSR, INS1 and INS2.

The evolutionary history and the phylogenetic relationships pattern can be explored through the molecular approach like amino acid sequences [22]. We have shown seven functional proteins and their evolutionary relationship as well as evolutionary history through phylogenetic tree. For the modified phylogenetic tree with node and distance, we have used sparse function in our program, which generates matrices in the MATLAB sparse storage establishment. S =  sparse (A) alter a full matrix to sparse form by forcing out any zero elements. Assuming that, S is already sparse, S is being returned by sparse(S). S =  sparse(i, j, s, m, n, nzmax) utilize uses vectors i, j, and s to yield an m-by-n sparse matrix such that S(i(k),j(k))  =  s(k), with space allocated for nzmax nonzero. All vectors such as i, j, and s are having equal length and whatever the elements of s that are zero are discounted, along with the corresponding values of i and j. Whatever elements of s that have duplicate values of i and j are added together. S =  sparse (1:n,1:n,1) yields a sparse representation of the n-by-n identity matrix. Here DG is nothing but a directed graph, which differs from an ordinary or undirected graph [23] and in the above program it's a sparse matrix that stores the distance of the edges of the phylogram. In the distance of the phylogenetic tree, there are 12 nodes; we have used tree traversal programming in MATLAB simulator, which is more like a binary tree structure. Each edge is having some weights based on the distance from nodes. Edges which are broken (mentioned as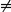 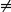) imply unknown distance between those two nodes. So, we have ignored those edges (assuming the distance as .50) while programming for the generation of the tree.

With the advancement of computer science, mapping proteins onto signaling pathways could be done easily, which would provide scientists an advantage of the rapid accumulation of proteomics information. Using computational methods, large-scale proteomics information can be analyzed faster and this way scientists could reduce the number of experiments required detecting the main interactive proteins of a pathway. The major interactive proteins can be validated *in vitro* as well as *in vivo* as a target protein for future drug development research.
